# Evolution of SLA-Based Al_2_O_3_ Microstructure During Additive Manufacturing Process

**DOI:** 10.3390/ma13183928

**Published:** 2020-09-05

**Authors:** Svyatoslav Chugunov, Nikolaus A. Adams, Iskander Akhatov

**Affiliations:** 1 Center for Design, Manufacturing & Materials, Skolkovo Institute of Science and Technology, 30/1 Bolshoi Boulevard, 121205 Moscow, Russia; i.akhatov@skoltech.ru; 2 Department of Mechanical Engineering, Technical University of Munich, Boltzmannstrasse 15, 85747 Garching, Germany; nikolaus.adams@tum.de

**Keywords:** microstructure, aluminum oxide, stereolithography, 3D printing, greenbody, debinding, sintering, porosity, computed tomography, FIB/SEM

## Abstract

Evolution of additively manufactured (AM) ceramics’ microstructure between manufacturing stages is a hardly explored topic. These data are of high demand for advanced numerical modeling. In this work, 3D microstructural models of Al_2_O_3_ greenbody, brownbody and sintered material are presented and analyzed, for ceramic samples manufactured with SLA-based AM workflow, using a commercially available ceramic paste and 3D printer. The novel data, acquired at the micro- and mesoscale, using Computed Tomography (CT), Scanning Electron Microscopy (SEM) and Focused Ion-Beam SEM (FIB/SEM) techniques, allowed a deep insight into additive ceramics characteristics. We demonstrated the spatial 3D distribution of ceramic particles, an organic binder and pores at every stage of AM workflow. The porosity of greenbody samples (1.6%), brownbody samples (37.3%) and sintered material (4.9%) are analyzed. Pore distribution and possible originating mechanisms are discussed. The location and shape of pores and ceramic particles are indicative of specific physical processes driving the ceramics manufacturing. We will use the presented microstructural 3D models as input and verification data for advanced numerical simulations developed in the project.

## 1. Introduction

Technical ceramics are modern materials with pre-engineered mechanical, thermal and electromagnetic properties demanded in multiple industrial applications. The most abundant and well-studied technical ceramic material is Al_2_O_3_ [1,2]. Typical applications of Al_2_O_3_ technical ceramics include aerospace, machinery, biomedical, energy (heat exchangers and gas turbines) [2,3] and others. While modern ceramic-made technological parts become more compact and sophisticated in shape, a shift from traditional manufacturing approaches to additive manufacturing (AM) became evident.

Many AM technologies were applied to ceramics shaping over the years. One of the most precise and robust methods of ceramics AM is a stereolithography-based approach (SLA) [4]. Based on our technical experience in the manufacturing of hundreds of industrial and scientific items, using the SLA technology, this method produces ceramic items from hundreds of micrometers in size to almost half of a meter, with a precision of 30–40 µm and high repeatability.

The SLA-based manufacturing procedure utilizes a paste-like material to 3D-print ceramic green parts. The paste consists of a fine (typical particle size ≤ 1 µm) ceramic powder and a viscous organic binder made of monomers/oligomers, photoinitiators sensitive to some light wavelengths, dispersants and plasticizers [5,6]. The paste is applied to a building platform in thin (20–50 µm) layers, where UV laser selectively polymerizes contours and shades areas. A 3D object is grown in a layer-by-layer process. The 3D-printed green part (a.k.a., greenbody) requires cleaning to remove the excess of the non-polymerized paste from external surfaces, internal channels and cavities. The cleaning process usually implies the use of a pressurized air-gun supplying a chemical agent that dilutes the unpolymerized paste and forces it out of the printed item. In some cases, mechanical tools are employed to assist with cleaning.

The cleaned green part needs to be slowly heated in an oven filled with air or inert gas to temperatures above 600 °C to facilitate the removal of the organic binder. The process is called “thermal debinding”; the processed item is called brownbody. Finally, the brown parts must be sintered in a kiln at high temperatures (often above 1000 °C) for densification.

Even though the SLA method allows advanced production of complex ceramic parts, the technology is prone to problems that need to be studied and solved. The SLA workflow consists of 4 technological stages—3D-printing, parts cleaning, thermal debinding and sintering. Each technological stage produces adverse effects on manufactured ceramic items. During the 3D-printing stage, random shifts could be expected in the printed parts that are floating over the layer of a viscous paste. Another adverse effect is the generation of microbubbles within a layer of ceramic paste. The microbubbles’ generation owes to air entrapment during paste application to the building platform. Modern 3D printers offer several approaches to reduce such effects but some defects still could be observed in the printed parts.

The 3D printed green part is susceptible to mechanical damage during the cleaning stage, due to the residual stresses developed in polymerized binders and mechanical load applied to local sections of the printed items with the cleaning equipment. Some items having long thin channels and internal cavities are impossible to clean out. The cleaning fluids cannot develop the necessary pressure to push unpolymerized ceramic paste out of such narrow voids. In some cases, the high pressure of the cleaning agent overcomes the mechanical strength of the greenbody and leads to damages.

The debinding stage is the most critical part of the entire workflow. Many fracturing events occur in processed parts during the thermal removal of the binder. Usually, the manufacturers understand that some intricate ceramic shapes cannot be produced with AM technology when they attempt to debind the parts. Upon successful removal of the binder from the greenbody, it becomes a brownbody part. The brownbody material features high porosity (~20–40%), interconnected voids of large volume and low mechanical strength, especially in the bending scenario.

The sintering stage leads to substantial material shrinkage, as well as to coalescence and reduction of small pores. The pores remaining in the sintered materials are descendants of large voids of the brownbody material. Pores are essential material components in porous ceramics [7,8,9,10], for applications like thermal insulation or gas filtration. For most structural applications, where material with utmost density is on-demand, pores need to be eliminated [11]. Sintering under a regular atmosphere often delivers 95–99% of theoretical material density [12]. Vacuum sintering or sintering under pressure help reaching low porosity values [13].

There are multiple attempts in the scientific community to study the fundamental aspects of the described problems and to optimize the manufacturing approaches targeting for defect-free technical ceramics. Among those, a series of experimentation [14,15,16,17,18] and advanced numerical modeling [19,20,21,22]. The development of numerical models for simulation of ceramic-related physical processes is closely related to the material’s microstructure. The distribution of ceramic particles, an organic binder and internal pores in the volume of the material, as well as, their geometrical and topological characteristics, are the critical parameters for initialization and validation of numerical models. In this work, we present the microstructure of Al_2_O_3_ ceramics at different stages of the SLA workflow. It is a preliminary step in the project focused on advanced numerical modeling of ceramics 3D printing, debinding and sintering processes.

## 2. Materials and Methods

### 2.1. Samples Manufacturing

There were 30 Al_2_O_3_ samples 3D-printed using Ceramaker 900 3D-printer (3DCeram, Limoges, France) to investigate the changes in the microstructure of ceramic parts through the stages of the SLA workflow. The samples were produced from the genuine Al_2_O_3_ ceramic paste, purchased from 3DCeram (Limoges, France). The paste consists of an acrylate-family monomer/oligomer composition, a photoinitiator sensitive to the light with a wavelength of 355 nm, a plasticizer, a dispersant and ~63 vol.% of α-phase Al_2_O_3_ ceramic powder. The ceramic powder is characterized by a wide particle size distribution curve. The average particle size is 1.05 µm. The majority of the particles have a size of 0.5–2 µm. A rare occurrence of large particles (up to ~5 µm) is observed in the paste [19].

The layer thickness during the 3D printing was 50 µm, the in-layer resolution was 45 µm. The average size of the produced samples was 10 × 6 × 5 mm. The greenbodies were cleaned with a liquid solvent, provided by 3DCeram. According to the manufacturer, the solvent dilutes the unpolymerized ceramic paste, efficiently reducing its viscosity to aid with paste removal and produces no adverse effects to the polymerized material. The ten samples were characterized in the green state with laboratory methods.

The remaining 20 samples were debinded in the air in Kittec CLL-15 (Kittec, Germany) furnace, using the thermal curve, provided by the printer’s manufacturer as a part of a commercial 3D printing package. The maximum temperature was 600 °C. The maximum heating rate of 12 °C /hour was used for non-critical sections of the temperature curve and up to 6 °C /hour for the critical sections.

During the debinding process, all organic components were removed from the ceramic green parts, while ceramic grains remained intact. For the investigated material, the thickness of greenbody specimens suitable for thermal debinding is usually limited to about 3 mm for parts with complex cross-sections and 5 mm for flat parts. This limitation is related to the release of large portions of gas in the volume of the material when the thermal decomposition of the organic binder is happening. In thick samples, the gas cannot efficiently escape from the bulk material. It leads to internal pressure build-up and subsequent fracturing of the debinded part. The 20 greenbody samples were thermally debinded and checked for possible fractures with Zeiss Axio Scope A1 optical microscope (Zeiss AG, Oberkochen, Germany). Laboratory characterization was applied to ten brownbody samples.

The remaining ten brownbody samples were sintered in ThermConcept HTL 20/17 (ThermConcept, Germany) kiln in the air at 1700 °C for 1.5 h. The maximum heating rate was 3 °C/min. The sintering curve was provided by the printer’s manufacturer. Since the ceramic items shrink during sintering, the average size of the sintered samples reduced to ~8.7 × 5.3 × 4.3 mm. The ten sintered samples were characterized by laboratory methods.

### 2.2. Characterization of Samples Microstructure

Laboratory characterization methods applied to greenbody, brownbody and sintered samples included—samples weighing with A&D HR250-AZG analytical scales (A&D Company Limited, Japan, the precision of 0.1 mg), measuring samples’ outer dimensions with Topex dial caliper (Grupa Topex, Warsaw, Poland, precision 0.02 mm), Computed Tomography (CT), Scanning Electron Microscopy (SEM) and Focused Ion-Beam SEM (FIB/SEM). The density of the samples was calculated from the samples’ mass and volume. Since the samples had a rectangular shape with flat walls and uniform cross-section, the measurements were made with low error.

The SEM and FIB/SEM methods were applied to building detailed 3D models of material microstructure. These methods are destructible—one sample of each type was fragmented to reveal the broken surface for SEM imaging. For FIB/SEM analysis, small portions of the samples were repeatedly milled off with a Ga ion beam and imaged with SEM.

Computed tomography was carried over with V|tome|x M300 tomograph (Baker Hughes, Huston, TX, USA) at a scanning resolution of 2 µm/voxel. The CT results contained a stack of thousands of 2D greyscale images in Tagged Image File Format (TIFF). Each 2D image represented spatial density (in fact, the z-number or X-ray attenuation) distribution at particular locations in the scanned material volume. 3D models of material microstructure were assembled via combining the 2D images into vertical stacks. The image processing of 3D models was made with Avizo v9.0 (FEI—ThermoFisher Scientific, Waltham, MA, USA) software. The 3D models were corrected for X-ray beam hardening effects and smoothed with non-local means filter. The edges of constituents in the models were sharpened with unsharp masking. Sub-volumes of 1 × 1 × 1 mm size were extracted from the CT models for analysis. Histogram-based thresholding was used to segment out the entities of interest. “Label analysis” tool of Avizo software was used to get geometrical characteristics of the segmented entities.

In CT images, ceramic particles, small pores and organic binder are homogenized at micro-scale—below the resolution limit of the tomograph device. Such small constituents could not be resolved in the 3D models. At scanning resolution of 2 µm/voxel, only objects >4–5 µm in size could be successfully resolved. To account for this limitation, the main focus of microstructural analysis of CT data was set on the segmentation of large pores (size higher than 4–5 µm) that have a round shape. Such pores are believed to be attributed to air bubbles entrapped in the paste. The pores were segmented out for greenbody, brownbody and sintered ceramic materials. These pores were plotted in 3D to demonstrate the typical distribution of large (size higher than 4–5 µm) pores in ceramic samples at different stages of the additive manufacturing workflow. Additionally, a size distribution histogram was built for those pores, using handmade code in Matlab (MathWorks, Natick, MA, USA).

SEM imaging, using FEI Versa 3D (FEI—ThermoFisher Scientific, Waltham, MA, USA) dual-beam analytical system, was applied to broken surfaces of the samples to capture the typical landscape of the greenbody, brownbody and sintered ceramic materials. The scanning resolution was 9 nm/pixel. The imaging was made using the Circular BackScatter (CBS) detector of the SEM device. Greenbody and brownbody samples demonstrated uniform properties over the studied regions. Hence, random locations were selected at the broken surfaces for FIB/SEM examination. For the sintered sample, the spots with possible porosity were identified at the broken surfaces for FIB/SEM examination. The use of a FIB/SEM instrument at non-porous locations, that is, inside grains, of the sintered sample yields a 3D cube, almost uniformly filled with noisy grey color—no distinct features, such as pores or grain boundaries, could be identified. FIB/SEM imaging was carried over with the FEI Versa 3D dual beam analytical system with an in-slice resolution of 3 × 4 nm and a slice thickness of 30 nm. Cubes of 10 × 10 × 10 µm were acquired. After alignment, cropping, filtration and edge defects removal, the 5 × 5 × 5 µm models were prepared for greenbody, brownbody and sintered samples.

The FIB/SEM results were represented by a stack of hundreds of 2D TIFF greyscale images. When assembled into an image stack, they constitute a 3D model of the scanned sample volume. The data were processed with Avizo v9.0 software. The images were aligned in the stack, using features recognition algorithms of Avizo—this step reduced the drift effects introduced by SEM detectors into images, due to thermal drift and charge accumulation during long scanning. The aligned stack was cropped, the images were processed with Fast Fourier Transform filters to remove stripes and strikes, generated by ion beam during material removal and electron beam due to charge accumulation at the fresh slices. The filtered images were smoothened with Non-Local Means filter and sharpened with Unsharp masking. Watershed segmentation algorithm was applied to the greyscale images to segment out ceramic particles, an organic binder and pores within the volume of the sample.

The ceramic particles, organic binder and pores were 3D plotted in separate images to demonstrate the typical spatial distribution of these phases in the volume of ceramic samples at different stages of the additive manufacturing workflow. Because pores in the studied materials (especially for FIB/SEM results) have not round, complex shapes with multiple percolating regions, volume distribution histograms were built to analyze the extent of the porous systems within the samples. An interconnected porous system might have linear dimensions similar to those of the entire sample but a tiny volume. Under such circumstances, the use of pore volume instead of pore size in the histograms is rational.

## 3. Results

**Figure 1:**
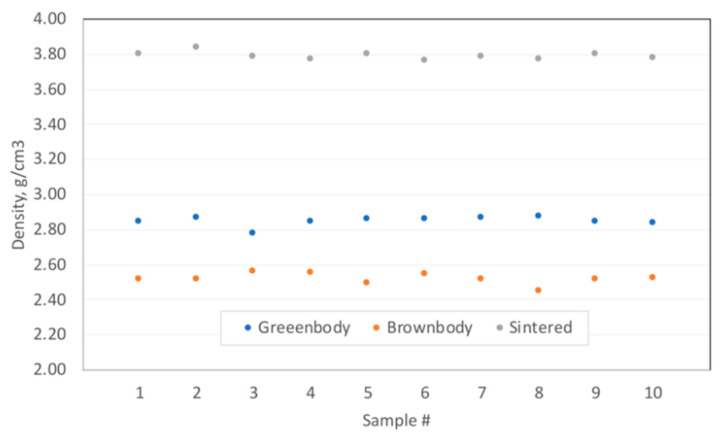
The density of the samples manufactured with stereolithography-based additive manufacturing workflow. The demonstrated samples (10 for greenbody, 10 for brownbody and 10 for sintered material) were evaluated independently. There are no inter-relations between the shown data-points.

Samples of greenbody, brownbody and sintered Al_2_O_3_-based materials were characterized with conventional methods of density estimations (Figure 1) and with methods of microstructural analysis. Based on the measurements of samples’ mass and dimensions, the following average apparent density values were calculated—greenbody—2.85 g/cm^3^, brownbody—2.52 g/cm^3^ and sintered ceramics—3.79 g/cm^3^. The standard deviation was 0.0273 g/cm^3^, 0.0312 g/cm^3^ and 0.0225 g/cm^3^, correspondingly. The ceramic powder used in the paste had a density of 3.99 g/cm^3^. Since brownbody and sintered samples contain only ceramic material and voids, their average porosity was determined directly—brownbody—37.3% and sintered ceramics—4.9%. The porosity of the individual samples was in the range of 35.9–38.6% for brownbody and 3.8–5.6% for sintered parts.

The greenbody samples contain not only ceramics but also organic binder; hence, their composition is harder to estimate. We assume that the average ceramic content of the greenbody samples is the same (62.7 vol.%) as for the brownbody ones since debinding induces no shrinkage. Based on the results of the CT and FIB/SEM study, presented below, the overall porosity of the greenbody sample is assumed as 1.6%. Then the calculated composition of the greenbody material is 62.7 vol.% of ceramic powder, 35.7 vol.% of organic binder and 1.6% of pores.

**Figure 2:**
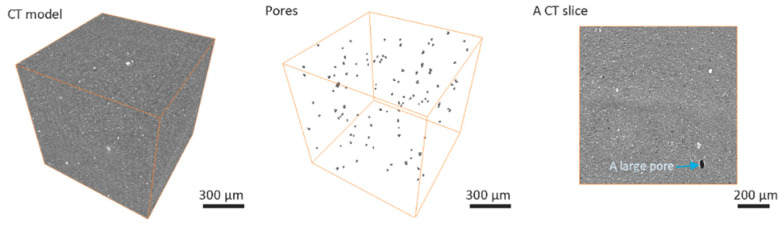
Computed tomography results for Al_2_O_3_ greenbody ceramic sample, manufactured with stereolithography-based additive technology, there are shown the original greyscale 3D image acquired with computed tomography (CT), the segmented round pores and a typical CT slice.

**Figure 3:**
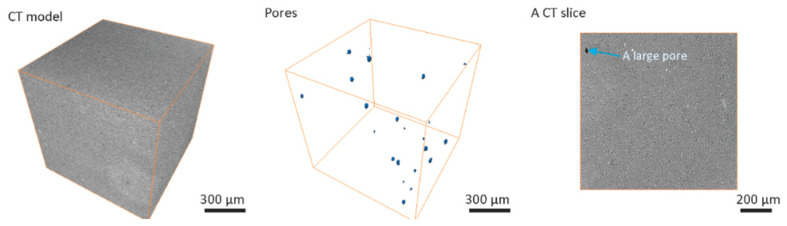
Computed tomography results for Al_2_O_3_ brownbody ceramic sample, manufactured with stereolithography-based additive technology, there are shown the original greyscale 3D image acquired with computed tomography (CT), the segmented round pores and a typical CT slice.

**Figure 4:**
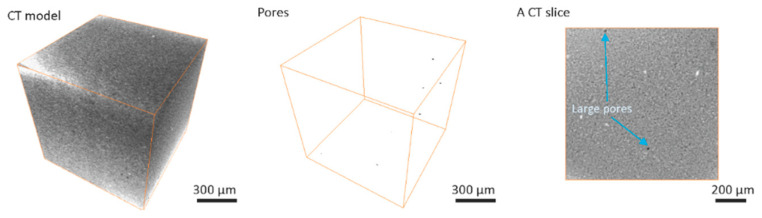
Computed tomography results for Al_2_O_3_ sintered ceramic sample, manufactured with stereolithography-based additive technology, there are shown the original greyscale 3D image acquired with computed tomography (CT), the segmented round pores and a typical CT slice.

Computed tomography results for greenbody (Figure 2), brownbody (Figure 3) and sintered (Figure 4) ceramic samples revealed the presence of relatively large (up to 32 µm) round-shaped pores in all types of samples. Examples of such pores in the CT results are indicated with blue arrows in Figure 2, Figure 3 and Figure 4. Image processing and segmentation of large round pores are relatively easy for greenbody (Figure 2) and sintered samples (Figure 4). In these materials, the large round pores can be distinguished from other objects by their size. For brownbody sample, the application of pore segmentation algorithm yields not only round pores originated from air bubbles but also multiple curved pores that emerged after organic binder was removed from the greenbody. The curved pores have a size similar to the round ones but their origin is different. The curved pores were filtered out during image processing of CT results. Only pores originated from air bubbles were kept for consistency with other CT models (Figure 3).

The overall number of round pores is the largest (115 pores per 1 mm^3^) in the greenbody sample. The brownbody sample contains a lower number of pores (22 pores per 1 mm^3^) because many round pores existed in the greenbody material merged with new voids, emerged from organic binder removal and became a part of a vast interconnected void system, rather than single isolated pores. These pores were filtered out due to their shape was not round anymore. It was not possible to determine whether they originated from air bubbles. The round pores remaining in the brownbody demonstrated greater size when compared to the ones in the greenbody material. This effect is explained via coalescence of the original round pores with the voids remained after binder removal. In the sintered sample, the number (9 pores per 1 mm^3^) and the size of round pores were low.

The smallest pores, identified with CT method for all types of samples, were 4–5 µm (2 × 2 × 2 voxels) in size. The largest pore size observed in the samples was 22 µm for greenbody, 32 µm for brownbody and 17 µm for sintered samples. The observed pores were smaller than the size of the laser beam (40 µm). They could potentially be generated from local evaporation of volatile components of organic binder upon laser action. The CT results indicate that no bubbles greater than the size of the laser beam were entrapped in the material during SLA-based 3D printing—3D printer’s mechanization, designed for removal of large (100 µm–1 mm) bubbles, demonstrated efficient work.

The overall contribution of bubble-originated round pores to the total porosity of the samples is 0.018% for greenbody sample, 0.01% for brownbody sample and 0.0002% for sintered material. The rest of the material porosity consists of pores having either smaller size or not round shape (for brownbody sample only).

Besides the pores, a few bright inclusions were observed in Figure 2, Figure 3 and Figure 4. The inclusions could be the traces of ZrO_2_ particles introduced to the ceramic paste during the homogenization procedure at the paste manufacturer’s facility. The homogenization often employs a planetary ball milling with zirconia jars and zirconia mixing balls. Another possible source of foreign particles might be the procedure of powder milling to the micrometer size fraction at the powder manufacturing facility. The foreign particles are well discernible in CT images due to the differences in their X-ray attenuation, but, in SEM images, they are hard to find, since their shape and electron scattering behavior resemble that of the base ceramic powder. Such inclusions might be hard for studying with Energy Dispersive Spectroscopy (EDS) method due to their scarcity in material’s volume and the absence of prominent features distinguishable in SEM images, that are often used as a primary navigation tool for EDS applications.

**Figure 5:**
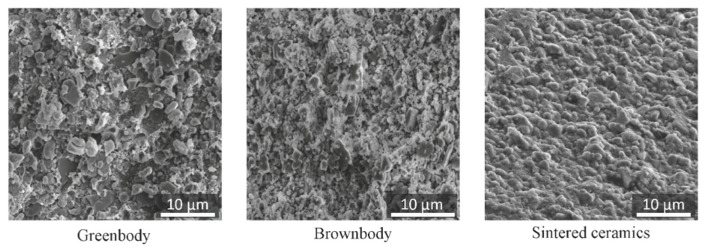
Scanning electron microscopy (SEM) images of Al_2_O_3_ ceramic samples, manufactured and processed with stereolithography-based additive technology.

The typical SEM images of a broken (not polished) surface of greenbody, brownbody and sintered ceramic material are shown in Figure 5. Ceramic particles, perceptible in greenbody and brownbody samples, have the shape of flakes with size ranging between 100 nm and 4 µm. In the greenbody sample, these particles are embedded into a polymer matrix. In the brownbody sample, there is no polymer in the structure, which leads to intensive charge accumulation on the surface of ceramic particles during SEM imaging. The sintered sample demonstrates a surface where ceramic particles are fused as a result of thermal processing. Small pores and fractures that were visible in greenbody and brownbody samples could not be identified in the sintered material, which is an indication of proper densification.

The charge accumulation in the sintered ceramic material due to FIB/SEM operations is so high that it causes the drift of electrons and defocuses SEM images of scanned slices. When the scanned material volume has a size of 5 × 5 × 5 µm, the defocusing is tolerable. When the size increases to 10 × 10 × 10 µm the degree of exposure of the material to ions and electrons increases and charge accumulation spoils the process. The standard imaging process of the dual-beam analytical system needs to be modified to lower charge accumulation effects at the surface of the imaged slices. In particular, the removal of the material surrounding the studied volume is necessary, the decrease of scanning current is crucial and intermediate refocusing operations become important—4 to 5 refocusing stages were introduced into FIB/SEM imaging sequence for the presented models. Even after taking the extraordinary measures, the scanning of large volumetric 3D models of sintered ceramics might require 2–3 attempts at a professional electron microscopy facility.

**Figure 6:**
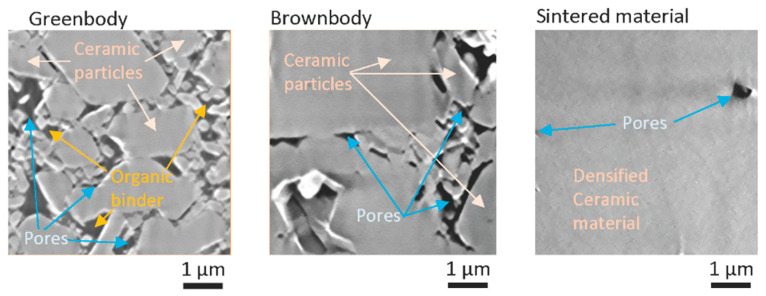
Typical slices acquired with Focused Ion-Beam SEM (FIB/SEM) characterization procedure for Al_2_O_3_ greenbody, brownbody and sintered ceramic samples, manufactured and processed with stereolithography-based additive technology.

**Figure 7:**
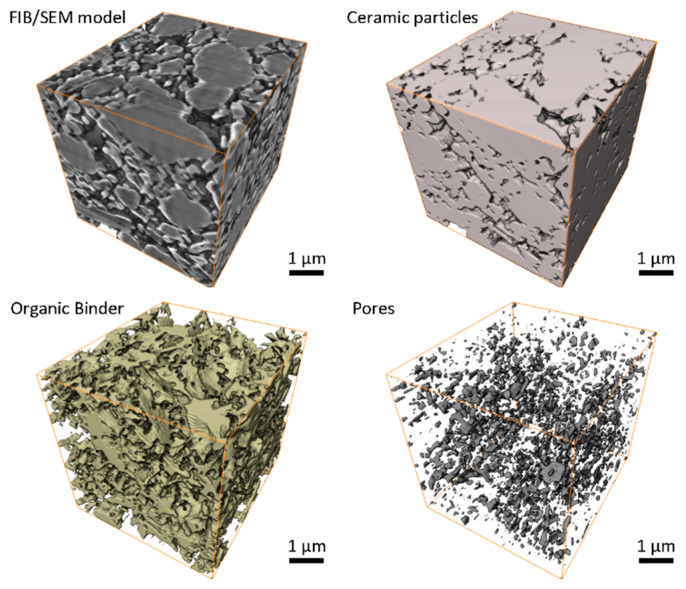
FIB/SEM-based 3D images of Al_2_O_3_ greenbody ceramic sample, manufactured with stereolithography-based additive technology, there are shown the original FIB/SEM greyscale 3D image and the results of image segmentation: ceramic particles, an organic binder and pores.

**Figure 8:**
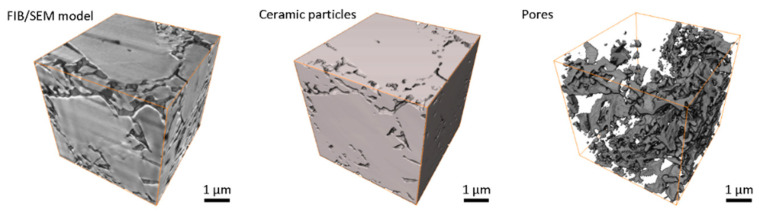
FIB/SEM-based 3D images of Al_2_O_3_ brownbody ceramic sample, manufactured and processed with stereolithography-based additive technology; there are shown the original FIB/SEM greyscale 3D image and the results of image segmentation: ceramic particles and pores.

**Figure 9:**
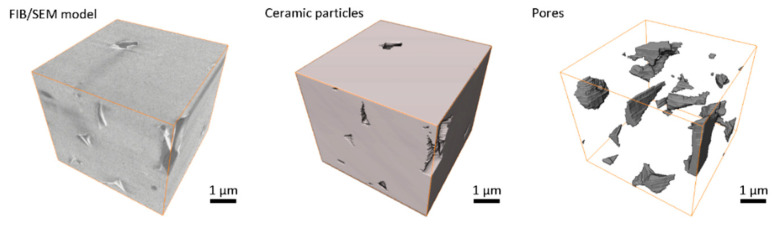
FIB/SEM-based 3D images of Al_2_O_3_ sintered ceramic samples, manufactured and processed with stereolithography-based additive technology; there are shown the original FIB/SEM greyscale 3D image and the results of image segmentation: ceramic particles and pores, remaining after sintering.

The FIB/SEM method allows building 3D models of material microstructure slice-by-slice. Since material components differently reflect and scatter electrons from SEM emitter, they could be differentiated and attributed to different material groups, such as ceramics, an organic binder or pores. Figure 6 shows an example of typical slices acquired with FIB/SEM instrument for greenbody, brownbody and sintered ceramic samples. Ceramic particles, an organic binder and pores could be distinguished in these images and segmented out into separate groups using watershed-based algorithms. Each 3D model typically contains 2–3 hundreds of such slices. Assembled into a stack, they allow visualization of materials microstructure in 3D (Figure 7, Figure 8 and Figure 9).

The FIB/SEM model of the greenbody sample represents the 3D microstructure of the material, including ceramic particles, an organic binder and pores (Figure 6). Due to the differences in electron scattering by ceramic particles, an organic binder and voids, these materials appear with different shades of grey color in the original greyscale FIB/SEM image (Figure 7). Separating these materials into isolated phases allows analysis of their distribution and interaction.

In the greenbody sample, ceramic particles are uniformly distributed over the entire sample volume. Some particles reach the size of 2–3 µm, while others are around 100 nm. The space between ceramic particles is filled with an organic binder. Some small spaces between adjacent ceramic particles remain dry, giving origin to complexly shaped pores. Additional small round pores were observed in the bulk of the binder. They have the size of tens to hundreds of microns. Most probably, they were generated due to air entrapment during paste homogenization procedure or resulted from volatile components saturation. The material groups in the greenbody sample have the following volume fractions—ceramic particles 71.0%, organic binder 27.4%, porosity 1.6%. The little pores in the binder bulk constitute a tiny part of the porosity. The significant portion of porosity is related to the dry zones between adjacent powder particles or to not wetted gaps between ceramic particles and organic binder. The former could be used as the quality estimates for the paste homogenization process and might serve for the mixing optimization. The latter might be an indication of the local spots where residual stresses generated from the binder polymerization led to the separation of the binder from ceramic particles—these regimes are essential phenomena for verification of numerical models of the SLA process.

The presence of pores in the SLA-produced greenbody allows faster relaxation of residual stresses generated during polymerization of the organic binder, decreasing the chances of greenbody damaging. In the debinding process, the pores of greeenbody may serve as reservoirs for gas accumulation and evacuation channels for gas diffusive flow during the thermal debinding process. This effect lowers the risks of greenbody damage when high pressure of gas develops in the material in the course of thermal decomposition of the binder. Additional analysis of pores’ locations and orientations in the greenbody’s volume could be useful for advanced numerical modeling. It might help to determine porosity contribution to build up and relaxation of residual stresses during photopolymerization of the organic binder, as well as the participation of the pores in gas filtration processes during thermal debinding.

The prime result of thermal debinding is the complete removal of organic binder from the volume of a green part. When the binder is thermally decomposed into gas and evacuated from the ceramic skeleton, the locations, previously occupied with the binder, become a part of a vast interconnected porous system (Figure 8). The shown microstructure of the brownbody sample could be used for verification of advanced numerical code, developed for detailed thermal debinding simulations.

It needs to be mentioned that the presented model contains two large (~4 µm in size) ceramic grains partially entering the analyzed volume, this is why the measured porosity constitutes only 11.7%, instead of the expectable 37%. The result demonstrated in Figure 8 is only one possible realization of the ceramic microstructure achieved with thermal debinding.

The ceramic sintering process leads to significant (15–20%) shrinkage of the brownbody material. As a result, most of the pores were eliminated—porosity decreased from 37 to 4–5%. Pores remained at the corners of neighboring grains. In most cases, the pores at the corners are relatively small in size (Figure 9). The 3D greyscale image contains some traces of intergranular boundaries but conventional segmentation approaches do not allow proper separation of ceramic grains—a special image processing method needs to be developed for this task. The shown microstructural image of the sintered material contains 2.1% of porosity. The shown 3D data for the sintered sample is a useful example of a 3D verification dataset to be used for advanced numerical simulation of the sintering process. It also has excellent potential to be used as input data for multi-scale numerical simulations of ceramics mechanical behavior.

**Figure 10:**
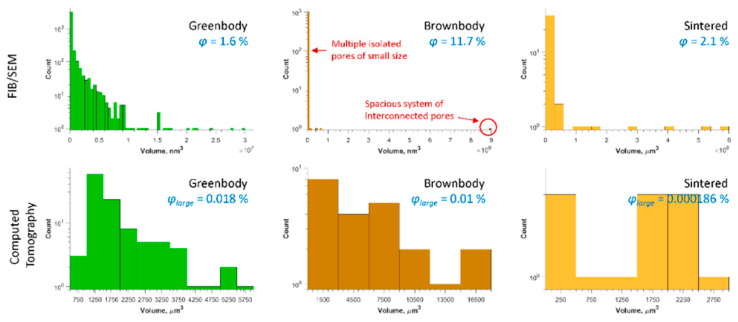
Volume distribution of single pores for FIB/SEM-based 3D images (top) and CT-based 3D images (bottom). The φ indicates the porosity of the samples, measured from FIB/SEM data. The φ_large_ indicates the contribution of round pores to the volume of the samples, investigated with the CT method.

The characteristics of the pores and porous systems, as the significant sources of heterogeneity in ceramic samples, are summarized in Figure 10 for all the demonstrated 3D models. For FIB/SEM models, there were multiple relatively small isolated pores in the greeenbody sample that contribute to the left side of the histogram—they are mostly originating from the dry regions in the vicinity of ceramic grains. A small number of pores is attributed to entrapped air nanobubbles. In the brownbody sample, the pores are connected to large porous systems. Some smaller (~10^7^ nm^3^) porous systems coexist with a large supersystem (~10^10^ nm^3^). They do not intersect within the studied subvolume, but might connect outside. The smaller porous systems contribute to the left side of the brownbody histogram, while the sizeable porous supersystem stands for a single entry at the rightest side of the histogram. There are 3776 single pores in the greenbody sample, while the brownbody sample contains 1003 single pores of higher volume. The sintered sample, studied with FIB/SEM instrument, contains 40 pores of the size 15 nm to 1 µm. The majority of the pores are located at the corners between contacting grains.

The large pores observed in CT models reach the size of 22 µm in the greenbody sample, though most of the pores have a size of 1–10 µm. Multiple voids were freed up of the organic binder after the debinding process. They led to an increase of the pore size (almost 1.4× or up to 32 µm) in the brownbody sample. Correspondingly, the histogram of the brownbody sample is shifted to the right, when compared to the greenbody results. After sintering, many large pores become eliminated—only a few occurrences of smaller pores were observed. The largest pore in the sintered material reaches 17 µm in size.

## 4. Discussion

The existence of a large number of pores in additively manufactured ceramic material at different stages of manufacturing might seem alarming. Nevertheless, Al_2_O_3_ ceramic samples, manufactured with SLA-based additive procedure and completely sintered, demonstrate physical-mechanical properties on par with traditionally manufactured technical ceramics. The size of the largest pores (32 µm), identified in this study, suggests that most of these defects are well below the resolution of SLA technology. Based on CT and FIB/SEM results, it might be concluded that the origin of the pores in additively manufactured ceramics is mostly attributed to two mechanisms—insufficient homogenization intensity (leads to dry regions, containing no binder, in the ceramic paste) and air entrapment (leads to the formation of bubbles of different sizes). When air bubbles are trapped during the homogenization procedure, they become small pores in the bulk of the organic binder. When air is entrapped during paste application to the building platform, round pores >100 µm are formed.

To remove the large (>100 µm) air bubbles during the paste application, the 3D printers might be equipped with special rollers, arranged along with the blades, that continuously mix the paste while it is being applied to the printing platform by a blade. This method provides a smooth layer of viscous paste over the printing platform. The removal of tiny nanobubbles from the organic binder might require vacuum-assisted homogenization of ceramic paste. At the same time, the reduction of pressure in the mixing chamber might initiate saturation of volatile organic components of the paste with subsequent formation of micro- and nanobubbles filled with volatile organic products. Therefore, the vacuum-assisted mixing approach might seem like a promising technology for the reduction of defects in greenbody but additional studies are required. Finally, the reduction of the “dry” zones in the greenbody might require intensification of the homogenization procedure. Even commercial ceramic paste could be additionally homogenized prior to the 3D-printing operations.

From another perspective, the presence of dry zones and nanobubbles in the organic binder might be a decisive factor that helps to reduce the residual stresses in the green body and assists gas evacuation during the debinding stage. When mechanisms of defect/pore formation and the role of pores in the material behavior under different regimes are well understood, special technical measures could be taken to reduce the adverse effects of the pores in additively manufactured technical ceramics.

The demonstrated two-scale 3D datasets allow a numerical analysis of a variety of essential physical processes, for example, the development of residual stresses in the greenbody as a result of ceramic paste photopolymerization; the thermal debinding of the green part including gas evacuation from sample’s interior, internal pressure build-up and mechanical simulations; the sintering of ceramic materials with the prediction of shrinkage anisotropy and shape distortions. Advanced processing and feature extraction from the presented microstructural models and their use for numerical simulations are the subjects of future work.

## 5. Conclusions

In this work, the novel results on the microstructure of a greenbody, brownbody and sintered ceramic samples were demonstrated in 3D and analyzed at microscale and mesoscale. The research was conducted as a part of the preparation procedure for advanced multi-scale numerical modeling of ceramics-related processes. The presented microstructural 3D datasets would be used as a part of input and verification data for numerical simulations. The analyzed samples represent different stages of SLA-based additive manufacturing workflow for ceramic items.

Mesopores and micropores were observed in the greenbody, the brownbody and the sintered ceramic samples. Based on the shape and location of the pores in the greenbody, two mechanisms of their origin were suggested—formation of dry (no binder wetted) zones and air entrapment due to not very intense homogenization of the ceramic paste. The pores in the brownbody sample are bigger, due to multiple voids formed and coalesced in the material when the organic binder was removed. The number of pores significantly decreases after sintering. The pore size gets much smaller. Advanced processing of the presented microstructural models will be continued in the scope of the development of numerical models for simulation of ceramics-related processes.
